# Modeling the cost of influenza: the impact of missing costs of unreported complications and sick leave

**DOI:** 10.1186/1471-2458-10-724

**Published:** 2010-11-24

**Authors:** Yiting Xue, Ivar Sønbø Kristiansen, Birgitte Freiesleben de Blasio

**Affiliations:** 1 Department of Biostatistics, Institute of Basic Medical Sciences, University of Oslo, Oslo, Norway; 2 Division of Infectious Disease Control, Norwegian Institute of Public Health, Oslo, Norway; 3 Institute of Health Management and Health Economics, University of Oslo, Oslo, Norway

## Abstract

**Background:**

Estimating the economic impact of influenza is complicated because the disease may have non-specific symptoms, and many patients with influenza are registered with other diagnoses. Furthermore, in some countries like Norway, employees can be on paid sick leave for a specified number of days without a doctor's certificate ("self-reported sick leave") and these sick leaves are not registered. Both problems result in gaps in the existing literature: costs associated with influenza-related illness and self-reported sick leave are rarely included. The aim of this study was to improve estimates of total influenza-related health-care costs and productivity losses by estimating these missing costs.

**Methods:**

Using Norwegian data, the weekly numbers of influenza-attributable hospital admissions and certified sick leaves registered with other diagnoses were estimated from influenza-like illness surveillance data using quasi-Poisson regression. The number of self-reported sick leaves was estimated using a Monte-Carlo simulation model of illness recovery curves based on the number of certified sick leaves. A probabilistic sensitivity analysis was conducted on the economic outcomes.

**Results:**

During the 1998/99 through 2005/06 influenza seasons, the models estimated an annual average of 2700 excess influenza-associated hospitalizations in Norway, of which 16% were registered as influenza, 51% as pneumonia and 33% were registered with other diagnoses. The direct cost of seasonal influenza totaled US$22 million annually, including costs of pharmaceuticals and outpatient services. The annual average number of working days lost was predicted at 793 000, resulting in an estimated productivity loss of US$231 million. Self-reported sick leave accounted for approximately one-third of the total indirect cost. During a pandemic, the total cost could rise to over US$800 million.

**Conclusions:**

Influenza places a considerable burden on patients and society with indirect costs greatly exceeding direct costs. The cost of influenza-attributable complications and the cost of self-reported sick leave represent a considerable part of the economic burden of influenza.

## Background

Influenza is a contagious respiratory illness with a long history of causing human morbidity and mortality. Despite extensive surveillance of seasonal influenza, its economic costs are hard to quantify for at least two reasons. First, only a small proportion of influenza infections are confirmed virologically, and a considerable proportion of cases registered as pneumonia or other diseases actually have influenza as the underlying cause [1]. There are many studies on the statistical methods for estimating excess hospitalization and mortality of influenza-attributable diseases [2-4], but few economic studies have incorporated the cost of these diseases. Second, in earlier studies that have estimated the indirect costs of influenza, self-reported sick leave has largely been ignored. To our knowledge, self-reported sick leave has only been estimated in small samples or through surveys [5,6], but not in the general population, and it has not been evaluated in combination with data on influenza-like illness or disease recovery curve.

Knowledge of the total costs of seasonal influenza is important because such data are crucial for studying the cost-effectiveness of influenza interventions (vaccination, isolation, etc.). In addition, the disease burden of seasonal influenza may be used to estimate the treatment capacities that society may need in case of a pandemic.

The aim of this study was to estimate the direct and indirect costs of seasonal influenza in Norway using Norwegian registry data on disease occurrence, use of health care services and sick leave certification. In this article, we introduce a novel method for estimating the costs of influenza based on influenza-like illness (ILI) data and sick leave register data. Using a variety of mathematical models, we estimated direct costs associated with non-specific diagnoses and all types of indirect costs, including self-reported absenteeism. Our results regarding seasonal influenza could form the basis for exploring the cost of a potential influenza pandemic.

## Methods

There are two types of costs of influenza: direct costs, which include the pharmaceutical costs and the medical service costs, and indirect costs, which stem from job absenteeism (productivity losses). A person infected with influenza will end up in one of the three outcomes: (1) ill but not medically attended; (2) ill with outpatient visit(s); and (3) ill with hospitalization (inpatients). Individuals in all three outcomes incur pharmaceutical and indirect costs, while only outpatients and inpatients incur medical service costs. Therefore, the three outcomes lead to four economic consequences: pharmaceutical cost for all three outcome groups, medical service costs for outpatients, medical service costs for inpatients, and indirect cost for all the three groups. For some of the costs, we estimated the number of events (hospitalization, sick leave days) and multiplied them by the relevant unit costs. For the others, we estimated them from aggregate cost data as reported in registries. In the end, we considered the uncertainty in data and performed a probabilistic sensitivity analysis. Our study used a prevalence rather than incidence approach to estimate cost-of-illness in part because influenza is not a chronic disease and in part because of data availability.

We assumed that each person could have at most one influenza episode during a season. We considered only symptomatic cases, since the asymptomatic cases do not contribute to the use of resources in the public health care system or to the job absenteeism.

### Data Sources

Data on influenza-like illness rates (ILI rates) by week during the 1998/99 through 2006/07 seasons were obtained from the Norwegian Institute of Public Health (NIPH). The ILI rate for a given week is the proportion of the number of general practitioner (GP) consultations in which an ILI diagnosis [7] is given out of the total number of consultations. Surveillance of influenza activity in Norway is based on weekly reports from 201 sentinels covering approximately 15% of all GP consultations in the country, and takes place from week 40 in one calendar year through week 20 in the next calendar year. No data are available on influenza episodes during the summer months. We therefore define an influenza season (year) as week 40 in one year through week 20 in the next year. Laboratory tests of influenza virus are only conducted for a minority of suspected influenza cases, but ILI rates have been proven to be a reliable proxy for influenza activity [8].

The attack rate *r_a _*refers to the percentage of the entire population with symptomatic influenza during an influenza season. This number is difficult to estimate since a large number of cases will receive only home care. NIPH estimates that the attack rate is between 5% and 15% in Norway (B. Iversen, personal communication, 16 June 2008).

We obtained from the Norwegian Patient Registry (NPR) weekly data on acute hospital admissions during the 1997/98 through 2005/06 seasons and data on average length of stay. The data included the number of hospital admissions in a given week with primary ICD-10 diagnoses of influenza (J10-J11) pneumonia (I20-I25), ischemic heart disease (J12-J18) and all-cause hospital admissions. We obtained diagnosis-related-group (DRG) data from NPR for all patients admitted to hospital with ICD codes influenza and pneumonia in the 2005/06 season. The DRG weights and the year-specific monetary value of a DRG point were acquired from a government publication [9]. We assumed that all patients have to consult their general practitioners before they are hospitalized.

Data on the weekly number of short-term doctor-certified sick leaves with ICPC-2 diagnoses of influenza (R80), other airway diseases and all-cause sick leaves for the 2000/01 through 2006/07 seasons were obtained from the Norwegian Labour and Welfare Service (NAV). In the following, "other airway diseases" means all R diagnoses except R80 in ICPC-2 and was considered as one category of disease. We merged week 52 and week 53, regarding them both as week 52 since week 53 has only a few days.

For pharmaceuticals used in relation to influenza-associated illness, the only data available are those on sales of Tamiflu. We obtained the data from the NIPH's prescription database [10]. For outpatient costs, we obtained from NAV aggregate monthly medical service costs of outpatients (GP visits, emergency room services and medical specialist visits) from October to May for the season 2005/06 with ICPC-2 code R80 (influenza) and with ICPC-2 code R81 (pneumonia).

All costs were measured in 2005/06 Norwegian kroner (NOK) and converted to US dollars (US$) using the exchange rate on 31 December 2005 (US$ 1 = NOK 6.75) [11]. Because we relied on aggregated registry data with no patient-level information, there was no requirement for an ethics approval for such a study under Norwegian regulations.

### Pharmaceutical cost

We examined two types of pharmaceuticals: Tamiflu, a medication that specifically targets at influenza, and other symptom-relieving medications that can be used for either influenza or cold symptoms. Tamiflu costs, including VAT, were based on redemption of prescriptions from all pharmacies in Norway. We used the average annual Tamiflu sales minus 25% VAT in years 2004--2008 as a proxy for the cost in 2005/06. To estimate the cost of other symptom-relieving medications, we surveyed pharmacies in Oslo, and found that their unit prices were approximately equal. Although a person with influenza may purchase none or several of the symptom-relieving medications, we initially assumed that an average of one pack per person was purchased, and later varied this assumption in the sensitivity analysis. The cost of other symptom-relieving medications was calculated as the product of the attack rate, the total population, the number of medications purchased per person (initially = 1) and the average price of these medications excluding VAT. Total pharmaceutical cost is the sum of the costs of Tamiflu and of other symptom-relieving medications.

### Cost of outpatient services

The aggregated monthly costs for influenza diagnoses were taken directly from NAV data for the 2005/06 season. To account for costs for pneumonia diagnoses with influenza as an underlying cause ("influenza-related pneumonia"), we multiplied NAV aggregate cost data for pneumonia diagnoses by the proportion of patients with influenza-related pneumonia. We assumed that this proportion was the same as the corresponding proportion calculated for pneumonia inpatients for the 2005/2006 season (See below, *Cost of inpatient services*). We further included the costs of influenza-related services provided by private clinics, which have been estimated to be approximately 20% of the costs in public sector (T. Sundell, personal communication, 31 March 2009).

### Cost of inpatient services

The medical service costs of inpatients were calculated as the product of the number of influenza-related hospitalizations and the unit cost of hospitalization. While we obtained the number of hospitalizations with influenza as primary diagnosis directly from NPR data, we had to estimate the numbers of hospitalizations of influenza-related pneumonia and of influenza-related other diseases, so-called excess hospitalization. We used three quasi-Poisson regression models to examine whether there is a correlation between the ILI rate and the number of hospitalizations for: 1) pneumonia, 2) ischemic heart disease, and 3) all causes, respectively. In each model, the dependent variable $\hat{Y}(w,s)$ was the predicted number of hospitalizations in week *w *of season *s*; it was explained by three covariates: the reported ILI rate, the week number *w *to capture weekly variations and the season number *s *to capture yearly variations:

(1) $$\hat{Y}\left(w,s\right)=\exp(\beta_{0}+\beta_{ILI}ILI(w,s)+\beta_{w}w+\beta_{s}s)$$

The model was fitted using the glm-package in the statistical software R version 2.7.1 (R Foundation for Statistical Computing, Vienna, Austria). Poisson regression, often used to analyze count data, assumes a data distribution in which the mean is equal to the variance. In our case, the variance and the mean of the data were not equal. Therefore we used quasi-Poisson regression in which this condition can be relaxed. We tested the model with the ILI rate of the same week and the ILI rate of the preceding week separately. Since Norway has five health regions (Eastern, Western, Southern, Middle and Northern), we also repeated the regressions for each of the regions separately.

The estimated number of excess hospitalizations of pneumonia or other diseases was calculated by week as the difference between the observed number of hospitalizations and the predicted number of hospitalizations given minimal influenza activity. The latter was obtained from Equation (1) by setting the influenza contribution (ILI rate) to a baseline while leaving the other parameters and covariates as they were. We define the baseline ILI rate as the mean of the lowest 10% of all ILI rates in the study period to capture the small year-round existing influenza activity.

The unit inpatient cost was calculated from Equation (2). Variable *j *designated the ICD code influenza or pneumonia when the patient is admitted into hospital and variable *i *designated the DRG diagnosis at discharge from the hospital. Let $\bar{C}(j)$ be the average hospital cost per patient admitted to hospital with ICD diagnosis *j *in the 2005/06 season. It was calculated as a weighted sum:

(2) $$\bar{C}(j)=\left(\sum_{i}\frac{m_{DRG}\left(i,j\right)}{n\left(j\right)}*w_{DRG}\left(i\right)\right)*U_{DRG}$$

where *n*(*j*) is the total number of patients with ICD *j*, *m_DRG_*(*i,j*) is the proportion of these patients with DRG code *i*, *w_DRG_*(*i*) is the DRG points for diagnosis *i *and *U_DRG _*is the monetary value per DRG point in 2005/06 season (US$4612 or NOK31130 [9]). Hospitalizations with unknown DRG codes were assigned 1 DRG point, assuming that they represented an average patient.

### Indirect costs

**Figure 1:**
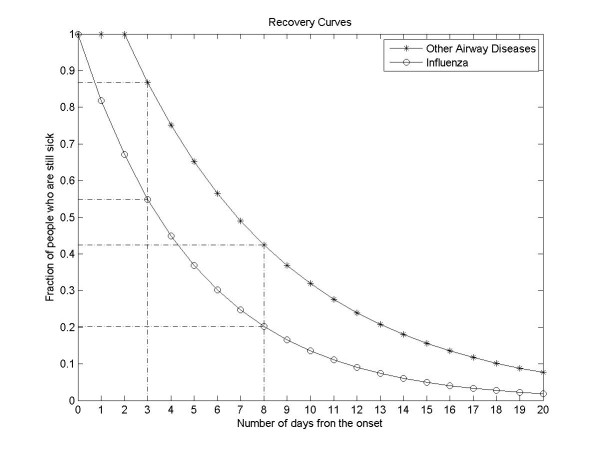
Recovery curves for influenza and "other airway diseases"

In Norway, doctors state a diagnosis on sick leave certificates while no information on diagnosis is available for self-reported sick leave. Data from NAV provided information on the number of doctor-certified sick leaves with influenza diagnosis, and sick leaves with "other airway diseases" diagnosis. We assumed that a fraction of the latter had influenza as the underlying cause. This fraction was estimated by means of quasi-Poisson regression in the same way as excess hospitalization (see Step 1 below). In addition to these doctor-certified sick leaves, employees may take sick leave for up to three days (eight days with some employers, see below) without a doctor's certificate, and this is denoted self-reported sick leave. If the person is still sick on the fourth (ninth) day, a sickness certificate from a doctor is needed in order to receive full salary. Assuming that there is a constant recovery rate that follows a negative exponential function of time from the onset of influenza (Figure 1), we estimated the total number and length of sick leaves on the basis of the number of doctor-certified sick leaves (Step 2). The indirect costs of self-reported and doctor-certified sick leave were then estimated from the total number of sick leave days and the average wage rate in Norway (Step 3).

Step 1: To estimate the number of non-influenza sick leaves which had influenza as underlying cause, we set up two quasi-Poisson regression models, same as Equation (1), to explore the relationship between the ILI rates and the number of sick leaves due to 1) "other airway diseases" and 2) all causes, separately. We calculated excess sick leave only when influenza was a statistically significant predictor in the regression model.

Step 2: In Norway, about 50% of the working population has an employer with "Agreement on Inclusive Working Conditions" (*Inkluderende Arbeidsliv* or *IA* a collective agreement between employer and employee). Employees with IA employers have the right to stay at home for up to eight calendar days with full salary without a doctor's certificate. About 40% of the working population is employed with non-IA employers and can stay at home for three calendar days with full salary without a doctor's certificate. The remaining 10% of the work-active segment is self-employed, and this group is not compensated financially during illness.

We assumed that if people with severe symptoms choose to go to work, their productivity is negligible and can be ignored.

The model was based on simulating the recovery curve for influenza and "other airway diseases". In this section, index *i *refers to influenza and index *a *refers to "other airway diseases". We assumed that recovery from illness occurs with a constant rate γ implying that the proportion *p*(*j*) of people who are still ill at the end of day *j *after the onset of symptoms is exponentially distributed *p*(*j*) = exp(-*j*γ). The mean recovery periods for influenza and "other airway diseases" were assumed to be 1/γ_*i *_= 5 days [12] and 1/γ_*a *_= 7 days, respectively. The recovery period for "other airway diseases" was 2 days longer than that of influenza because the infections in this case were complications to the primary influenza infection. Thus, we assumed that recovery starts at the end of the second day counted from the onset of symptoms; see Figure 1.

Based on the recovery curves we estimated the proportions of people who need doctor-certified sick leave for influenza (Figure 1): for non-IA employed *p_i_*(3) = 0.55 and for IA-employed *p_i_*(8) = 0.20. The corresponding fractions for other airway disease are *p_a_*(3) = 0.87 and *p_a_*(8) = 0.42.

The densities of the working population with non-IA and IA agreement are *ρ_nIA _*= 0.4 and *ρ_IA _*= 0.5, respectively. The term *N_i_*(*s*) refers to the total number of people with certified sick leave due to influenza in season *s*, and the values are obtained directly from the data. We use *N_i_*(*s*) to estimate the total number of people who were on influenza sick leave (including both doctor-certified and self-reported) in season *s, y_i_*(*s*):

(3) $$\begin{array}{l} y_{i}(s)\rho_{nIA}p_{i}(3)+y_{i}(s)\rho_{IA}p_{i}(8)=N_{i}\text{(s)}\\ \Leftrightarrow y_{i}(s)=\frac{N_{i}(s)}{\left(\rho_{nIA}p_{i}(3)+\rho_{IA}p_{i}(8)\right)} \end{array}$$

In the same way, we can estimate *y_a_*(*s*) from *N_a_*(*s*) where *y_a_*(*s*) is the total number of people who take sick leave for "other airway diseases" associated with influenza and *N_a_*(*s*) is the number of people who took doctor-certified sick leave of "other airway diseases" associated with influenza, which was obtained from the quasi-Poisson regression.

Step 3: We calculated the number of sick leave days from the number of sick leave people. The proportion of people with influenza who recover on day *j *is given by *r_i_*(*j*) = exp(-(*j*-1)*γ_i_*)-exp(-*jγ_i_*) for *j*≥1; these people will be away from work for *j *days. For people who recovered without visiting their doctors, we summed up the products of *r_i_*(*j*) and *j *days over the allowed 3 and 8 days (the first two terms in the parentheses in Equation (4)), and we assumed that the self-employed all recover within a maximum of 8 days (the third term in the parentheses in Equation (4)). The working days lost for people with certification is the sum of the average duration of sick leave with influenza *d_i _*and the average days prior to obtaining certification *α *(the 4th and the 5th term in the parentheses in Equation (4)). We have no information about when the certified sick leave is initiated within the approved time frame. Therefore, we simply set the onset of certified sick leave to be in the middle of the periods, and factors of *α_nIA _*= 2 and *α_IA _*= 4 days are added to the duration of sick leave *d*_*i*_. Finally, we multiply the expression by 5/7 since sick leave is counted as calendar days, including weekends, while there is no productivity loss from staying at home during the weekends. In conclusion, we get the total number of working days lost due to influenza,*D_i_*(*s*):

(4) Di(s)=5yi(s)7*(ρnIA∑j=13jri(j)+ρIA∑j=18jri(j)+ρse∑j=18jri(j)+(di+αnIA)ρnIApi(3)+(di+αIA)ρnIApi(8))

Similarly, for "other airway diseases", the loss of working days is:

(5) Da(s)=5ya(s)7*(ρnIA3ra(1)+ρIA∑j=16(j+2)ra(j)+ρse∑j=16(j+2)ra(j)+(da+αnIA)ρnIApa(3)+(da+αIA)ρnIApa(8))

The total number of working days lost related to influenza in season *s *is *D*(*s*) = *D_i_*(*s*)+*D_a_*(*s*). We multiplied *D*(*s*) by the mean daily wage for the 2005/06 season. An extra 40% was added to account for the value of productivity produced by workers but not returned to them as wage. This includes employer tax, payment for holiday and pension contributions in Norway [13-15].

To estimate the productivity loss from parents taking care of sick children, we assumed that each sick child requires two days of attendance at home from one parent [16]. The number of sick children was the product of the attack rate (mean 7.5%, range: 5%--10%) and the population under 10 years old. The value of one day's work was the same as that in the previous paragraph. We multiplied the number of parents affected, the number of days (assumed to be 2) and the value of one day's productivity and used the result as an estimation of the productivity loss from taking care of sick children.

### Sensitivity analysis

We explored the uncertainty of the data through probabilistic sensitivity analyses using Monte Carlo simulation. We varied the attack rate, the number of packs purchased in pharmacies per person, the outpatient cost, the percentage of outpatient cost in the private clinics, DRG points for hospitalizations and the number of hospitalizations (see above and Table 1 for details). We expressed the uncertainty in terms of 95% confidence interval for each of the cost components.

**Table 1:** Factors for estimating direct costs of seasonal influenza in the 2005/06 season

Direct cost		Mean	Distribution	Parameters	
Pharmaceutical cost	Attack rate (%)	7.50	Beta ^b^	α = 31.91	β = 393.5
	Cost per pack (USD)	7.4			
	Number of packs	1.00	Gamma ^c^	k = 6.83	Θ = 0.15
	Population (2005/06)	4 640 219			

Outpatient cost^a^	Adjusted influenza+pneumonia cost	3 415 900	Gamma ^c^	k = 25.00	Θ = 13.66e3
	Percentage in private clinics	20.00	Beta ^b^	α = 12.60	β = 50.40

Inpatient cost	Number of influenza hospitalization	446	Gamma ^c^	k = 43.02	Θ = 10.37
	Number of pneumonia hospitalization	1 382	Gamma ^c^	k = 35.48	Θ = 38.95
	Number of other hospitalization	890	Gamma ^c^	k = 148.6	Θ = 5.99
	DRG points for influenza hospitalization	0.59	Gamma ^c^	k = 25.00	Θ = 0.02
	DRG points for pneumonia hospitalization	1.47	Gamma ^c^	k = 25.00	Θ = 0.06
	DRG points for other hospitalization	1.00	Gamma ^c^	k = 25.00	Θ = 0.04

**Indirect cost**					

Sick leave	Recovery period for influenza (days)	5.00	Normal	σ = 0.50	
	Recovery period for other airway disease (days)	7.00	Normal	σ = 0.50	
	
	Percentage with IA employers	50.0	Normal	σ = 1.67	
	Percentage with non-IA employers	40.0	Normal	σ = 2.36	
	Percentage self-employed	10.0	Normal	σ = 1.67	
	
	Days prior to sick leave for non-IA employees	2.50	Uniform	range [2.00: 3.00]	
	Days prior to sick leave for IA employees	4.00	Normal	σ = 0.50	

^a ^Outpatient cost includes costs from GP visits, emergency room services and medical specialist visits.

^b^$f(x;\alpha ,\beta )=\frac{1}{B(\alpha ,\beta )}x^{\alpha -1}{\left(1-x\right)}^{\beta -1}$, where *B *is the Beta function.

^c^$f\left(x;k,\theta \right)=x^{k-1}\frac{e^{-x/\theta }}{\theta^{k}\Gamma (k)}$, where Γ is the Gamma function.

We summed the pharmaceutical cost (*P*), the outpatient cost (*O*) and the inpatient cost (*I*) to obtain the total direct cost, assuming independence between the three sub-costs. We calculated the variation of the total direct cost (*T*) as Δ*T*:

(6) $$\begin{array}{l} \;\Delta T\\ =(T_{up}-T_{low})\\ =\sqrt{{\left(P_{up}-P_{low}\right)}^{2}+{\left(O_{up}-O_{low}\right)}^{2}+{\left(I_{up}-I_{low}\right)}^{2}} \end{array}$$

where indices *up *and *low *refer to the 95% upper and lower confidence interval. The upper and lower bounds of the total direct cost were then calculated as *T_up/low _*= *T *± 0.5*Δ*T*.

In the indirect cost part, we varied three variables: the recovery period, the number of days before obtaining doctor's certificate and the distribution of people in IA, non-IA companies and the self-employed (Table 1).

## Results

### Pharmaceutical costs

With an attack rate of 7.5%, a population of 4.64 million (2005/06) and an average of one pack purchased per person in pharmacies, the pharmaceutical cost excluding Tamiflu was $2.58 million per year. During the 2004 through 2008 years, the average cost of Tamiflu was $285 600 (Table 2).

**Table 2:** Average costs of seasonal influenza (2005US$)

		Mean	Lower bound	Upper bound
			(95% CI)	(95% CI)
Pharmaceutical cost	Tamiflu	285 665		
	Other	2 575 322	691 010	6 401 872
	Sub-total	2 860 986	976 675	6 687 536

Outpatient cost	Public	3 415 900	2 215 418	4 758 469
	Private	683 180	243 696	1 475 125
	Sub-total	4 099 080	2 459 114	6 233 594

Inpatient cost	Influenza	1 213 563	584 232	2 295 357
	Pneumonia	9 369 162	4 126 879	17 933 785
	Other	4 104 548	2 248 653	6 932 703
	Sub-total	14 687 272	6 959 764	27 161 845

Direct cost		21 647 339	10 982 153	32 312 524

Indirect cost	Employees' own sickness	202 247 136	180 927 219	229 780 678
	Employees' children's sickness	28 763 862	28 763 862	28 763 862
	Sub-total	231 010 998	200 103 205	268 132 650

Total		252 658 337	220 086 635	298 627 517

### Costs of outpatient services

In the 2005/06 season, the cost of influenza outpatient services was $2.8 million. The average annual ILI rate in that season was 83% of the average annual ILI rate in the period 1998/99 through 2006/07 seasons. We assumed that the cost of that season also represented 83% of the cost of a season with an average annual ILI rate. Therefore the estimated cost of influenza outpatient service would be $3.37 million given an average annual ILI rate. In addition, 1 in 14 pneumonia hospitalizations was influenza-related, resulting in an influenza-related pneumonia cost of $44 000 ($53 000 with average annual ILI rate). The cost of private clinic services was estimated to be $683 000, contributing to a total outpatient cost of $4 million (Table 2).

### Costs of inpatient services

**Figure 2:**
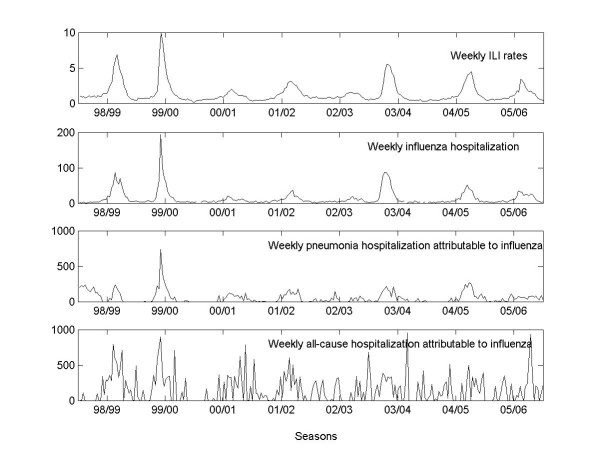
**Weekly influenza-like illness consultation per 100 consultations and influenza-related hospitalizations (The negative differences are set to zero in the figure but subtracted in calculation.)**

In the quasi-Poisson regression models, Equation (1), the ILI rate was found to be a significant predictor of acute all-cause hospitalizations (*P_total _*< 0.001) and pneumonia hospitalizations (*P_pneu _*< 0.001) at the national level. For ischemic heart disease, however, the association was not significant at the 5% significance level (*P_isch _*= 0.06). Similar patterns were present at regional level, although in the Middle and Northern health regions, ILI rate was a significant predictor of hospital admission for ischemic heart disease (*P_isch _*< 0.05). The weekly ILI rates and hospitalizations were plotted in Figure 2.

During the 1998/99 through 2005/06 seasons, the model estimated an average of 2700 influenza-related hospitalizations per year, among which 450 were registered as influenza, while 1400 were registered as pneumonia but having influenza as the underlying cause. The remaining 890 influenza-related hospitalizations had various other diagnoses that could not be identified (Table 3).

**Table 3:** Predicted numbers of influenza-related hospitalizations during the 1998/99 through 2005/06 seasons

	Hospitalization				**P&I**^**a **^**hospitalization**	Dominant
	Influenza	Pneumonia	Others	Total	**per 10**^**5 **^**persons**	**strain**^**b**^
1998/99	615	2312	1071	3998	66	H3N2
1999/00	717	2207	986	3910	65	H3N2
2000/01	229	552	531	1312	17	H1N1
2001/02	344	1087	1081	2512	32	H3N2
2002/03	210	659	675	1545	19	None
2003/04	651	1619	834	3104	50	H3N2
2004/05	417	1539	1097	3053	42	H3N2
2005/06	387	1080	845	2311	32	B

Mean	446	1382	890	2718	40	
SE	68	232	73	351	7	

^a ^Pneumonia and influenza

^b ^Data from Norwegian Institute of Public Health

The number of influenza-attributable hospitalizations varied from year to year with a threefold number of hospitalizations in the most severe year (1998/99) compared to the mildest year (2000/01) (Table 3). There was an annual average of 40 (SE = 7) influenza and excess pneumonia hospitalizations per 100 000 population during the study period (Table 3).

Among the patients admitted to hospital in the 2005/06 season, the cost for patients diagnosed with influenza represented a weighted average of 0.59 DRG points ($2721) while patients with pneumonia represented a weighted average of 1.47 points ($6780).

The total inpatient cost was on average $15 million per year during the 1998/99 through 2005/06 seasons while the total direct economic cost of influenza was $22 million (Table 2).

### Indirect cost

**Figure 3:**
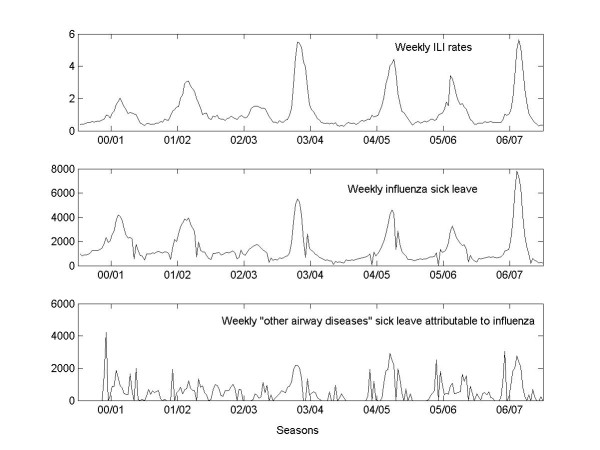
**Weekly influenza-like illness consultation per 100 consultations and influenza-related sick leave (The negative differences are set to zero in the figure but subtracted in calculation.)**

In the quasi-Poisson regression model, the ILI rate was found to be a significant predictor of sick leave for "other airway diseases". The ILI rate of the same week was a stronger predictor than that of the preceding week (*P_other-air-sick _*< 0.001). We did not find a significant association between the ILI rate and all-cause sick leave, either for the same week (*P_tot-sick _*= 0.21) or for the preceding one (*P_tot-sick-1 wk _*= 0.11). The weekly ILI rates and sick leaves were plotted in Figure 3.

On average there were 48 300 people certified sick with an influenza diagnosis each year and 11 000 people certified sick with influenza-associated "other airway diseases" (Table 4).

**Table 4:** Number of people with doctor-certified sick leave according to diagnosis

Season	Influenza	Other airway diseases
2000/01	60 768	7 187
2001/02	57 453	11 457
2002/03	34 694	6 867
2003/04	45 935	15 296
2004/05	44 694	12 095
2005/06	37 084	9 092
2006/07	57 340	14 758
Mean	48 281	10 964

SE	3 935	1 284

According to aggregate data from NAV, the average mean duration of doctor-certified sick leave was 7.2 days (range: 6.9 to 7.5) for influenza and 10.6 days (range: 10.1 to 10.9) for "other airway diseases" in the period 2000/01 through 2005/06.

The model results predicted that each year on average 104 700 people self-reported sick leave for influenza while 8 700 self-reported sick leave for "other airway diseases" attributable to influenza (Table 5). For influenza, the number of self-reported sick leaves represented 68% of the total sick leaves and 39% of the working days lost. For "other airway diseases" that is influenza-related, the corresponding numbers were 44% of the sick leaves and 22% of the working days lost.

**Table 5:** Sick leave according to type and diagnosis

		Number of people (95% CI)			Number of days (95% CI)		
		Mean	L-bound	U-bound	Mean	L-bound	U-bound
Influenza	Doctor-certified	48 281			343 839	327 159	360 272
	Self-reported	104 673	79 664	142 486	219 885	172 195	288 302
	Both types	152 954	127 945	190 767	563 724	513 486	632 653

Other airway disease	Doctor-certified	10 964			102 346	98 390	106 254
	Self-reported	8 717	7 234	10 476	28 460	23 572	34 254
	Both types	19 681	18 198	21 440	130 806	124 227	137 867

Both	Doctor-certified	59 245			446 185	425 549	466 526
	Self-reported	113 390	86 898	152 962	248 345	195 767	322 556
	Both types	172 635	146 143	212 207	694 530	621 316	789 082

Having sick children		49 389	32 926	65 852	98 777	65 852	131 703

Total		222 024	179 069	278 059	793 307	687 168	920 785

During the 2000/01 through 2006/07 seasons, there were on average 172 600 sick leaves due to influenza per year, including 153 000 sick leaves for influenza and 19 600 sick leaves for "other airway diseases" attributable to influenza (Table 5). The total number of people who were absent from work represented approximately 8% of the total workforce (approximate 2 million). In total, 793 000 working days were lost due to influenza, representing a total productivity loss of $231 million.

Parents taking care of sick children represented an estimated number of 98 800 working days lost, or a productivity loss of $29 million, approximately 13% of total productivity loss.

The total cost of influenza was $253 million per year, ranging from $220 to $299 million depending on severity (Table 2).

### Sensitivity analysis

In the Monte Carlo simulation, the total pharmaceutical costs varied from $0.98 million to $6.69 million due to the uncertainty in attack rate and the number of symptom-reducing medications purchased per person. Outpatient costs varied from $2.5 million to $6.2 million while inpatient costs ranged from $7 million to $27 million (Table 2).

Mean direct costs were $22 million with 95% confidence intervals of ($11 million, $32 million), and mean indirect costs were $231 million (95% C.I., $200 million, $268 million) (Table 2).

## Discussion

Our results indicate that seasonal influenza in Norway costs approximately $250 million per year, of which the indirect costs represent approximately 90%. Using national databases, we employed novel methods to estimate the economic cost of seasonal influenza. First, we modeled total excess hospitalizations attributable to influenza in order to estimate hospitalizations that did not list influenza as the diagnosis. Second, we developed a model to predict the cost of self-reported sick leave by combining data on GP-certified sick leave with disease recovery curves. Absence from work without GP certification is difficult to estimate and, to our knowledge, has only been estimated based on small samples and surveys in earlier studies [5,6]. The novel method presented here is general and may be adapted to estimate self-reported sick leave for other infectious diseases and in other countries.

Our results on influenza-related sick leave suggest that the number of people who took self-reported sick leave accounted for approximately 65% of the total number of people who took sick leave and that the number of days of self-reported sick leave accounted for 36% of the total number of sick leave days. Parental sick leave represents approximately one-tenth of the total indirect cost. The average duration of doctor-certified sick leave days was 7.2 days (range: 6.9 to 7.5) during the 2000/01 through 2005/06 seasons, which is slightly longer than the findings in studies in other European countries [6]. In Norway, employees receive full salary during sick leave, and this may explain longer spells of absenteeism in Norway.

According to our analyses, only 1 in 6 influenza-attributable hospitalizations had influenza as the diagnosis. 1 in 14 pneumonia hospitalizations had influenza as underlying cause and these hospitalizations accounted for approximately half of the influenza-attributable hospitalizations. We expected some of ischemic heart disease hospitalizations might also be caused by influenza. However, the national level results did not support this at a 5% significance level. In the Northern and Middle regions, however, there was a significant correlation between hospitalizations for cardiovascular disease and ILI rates, but we do not have a clear explanation for this difference.

We validated our method by comparing our results to the results produced by the software program *StatFlu *[17]. *StatFlu *was developed by the Swedish Board of Health and Welfare to estimate hospital load in the event of pandemic influenza. Using Norwegian demographic data, the *StatFlu *program estimated 1900 annual hospitalizations with an attack rate of 5%, and twice as many with an attack rate of 10%. Our results of approximately 2700 annual hospitalization per year were consistent with an attack rate between 5% and 10%. This suggests that that our assumption of an attack rate of 7.5% for seasonal influenza in Norway may be reasonable. Our finding that approximately 8% of the working population took influenza-related sick leave also supports this assumption.

We did not include the cost of lost lives in the economic analyses. The majority of seasonal influenza fatalities occurred to people over 65 years of age [18], and consequently the value of permanently lost productivity is minor. However, other studies have found that lost lives represent a considerable economic burden of influenza [19,20], and therefore our study may provide a conservative estimate of the economic burden of seasonal influenza.

Historic data on pandemics in the 20th century indicate that attack rates range from approximately 15% to 30% [21], representing a 2-fold to 4-fold increase in the attack rates compared to the 7.5% we assumed for seasonal influenza in this study. The results of the study may be used to shed some lights on the cost of an influenza pandemic. For example, assuming a pandemic attack rate of 3 times the seasonal average, we simply scale up the seasonal influenza cost by a factor of 3. In addition, there is a cost for the Norwegian government of stockpiling Tamiflu for pandemic influenza (K. Sælensminde, personal communication, 10 November 2009). The estimated total cost for pandemic influenza is approximately $800 million. With attack rates of 2 and 5 times the seasonal average, the estimated costs are $550 million and $1300 million, respectively. On the other hand, the Norwegian government has spent NOK 600 million ($88 million) on pandemic influenza vaccine (K. Sælensminde, personal communication, 10 November 2009), and with administrative costs included, this amounts to approximately $100 million. Hence, if the vaccine can prevent approximately 12% of the influenza cases in a susceptible population, it will be cost-effective or even cost saving unless there are severe side effects.

This pandemic cost calculation is highly simplistic, neglecting changes in the age-specific attack rates, severity of influenza-related diseases and potential costs from disruption of commerce and societal functions. In addition, the permanent productivity losses from mortality of young people may be considerable. Therefore, the pandemic influenza costs that we present above are clearly underestimated. However, the novelty of the virus coupled with changes over time in social structure and progress in medical technology made it difficult to determine the impact from historic data.

This study has several limitations. First, there are no registry data available for over-the-counter drugs for influenza. Second, the ILI reports from GPs, the hospital diagnoses and the sick leave diagnoses cannot be validated, and some might be inaccurate. Third, the numbers of excess hospitalizations depend on how the ILI baseline rate without influenza outbreaks is defined. Here, there is no agreement on the best research practice.

## Conclusion

Accurate estimates of the economic burden of influenza are essential for designing appropriate policies in public health. Standard measures of the societal costs of influenza generally omit the costs of influenza-related disease for which influenza is not the diagnosis and of self-reported sick leave. Our results indicate that including health care costs of influenza-related diseases may increase total costs by approximately 30% and that including the costs of self-reported sick leave may increase total costs by 40%. Based on these findings, vaccination for pandemic influenza may be cost-effective even at low prevention rates.

## Abbreviations

ICD: International Classification of Diseases; ICPC: International Classification of Primary Care, Second edition

## Competing interests

The authors declare that they have no competing interests.

## Authors' contributions

Study conception and design: YX, BFB. Acquisition of data: ISK, BFB. Estimating hospitalization and modeling sick leave: YX, BFB. Estimating economic costs: YX, ISK. Drafting of manuscript: YX. Critical revision: ISK, BFB. All authors read and approved the final manuscript.

## Pre-publication history

The pre-publication history for this paper can be accessed here:

http://www.biomedcentral.com/1471-2458/10/724/prepub
